# Approximation, Mad Men and the Death of JFK

**DOI:** 10.1007/s10699-016-9517-4

**Published:** 2016-12-24

**Authors:** Stella Bruzzi

**Affiliations:** Department of Film and Television Studies, University of Warwick, Coventry, CV4 7AL UK

**Keywords:** *Mad Men*, President Kennedy, JFK assassination, Zapruder, Representing history

## Abstract

In this article I take the US television series *Mad Men* (2007—present) as an exemplary ‘approximation’, a term I adopt to signal the way in which certain texts construct a changeable, fluid ‘truth’ resulting from collisions, exchange and dialectical argument. Approximations are layered, their formal layerings mirroring a layered, multifaceted argument. *Mad Men* integrates and represents real historical events within a fictional setting, and act that suggests that an event or action can never be finished, fixed and *not* open to reassessment. Specifically, this article examines ‘The Grown Ups’, Episode 12 of Season 3, which charts the events of 22 November 1963, the day Kennedy was assassinated. Although we might be able to bring to mind the images and conspiracy theories that have been made available since (such Abraham Zapruder’s 8 mm home movie footage of the assassination), these images were not available at the time. *Mad Men* as a series always strives to represent its historical milieu as authentically as possible, so the characters re-enact 22 November 1963 as authentically as possible by watching only what was on television that day (the news bulletin, Walter Kronkite’s announcement that Kennedy is dead). The contemporary backdrop to these events, including the resonances of ‘9/11’ through *Mad Men*, inform and collide with the authenticity on the screen.

As represented by this mathematical symbol ≈, approximations are values or quantities that are similar, almost equivalent, but never exactly the same. The term ‘*approximation’* encapsulates the belief that truth results from non-linear thinking and logic, that it is arrived at creatively; that such reassessment and continued dialogue can be a political as well as a creative act, so that events, texts, actions or individuals can be studied anew—and judgements of them potentially radically altered. For thinking about *Mad Men*’s representation and use of real historical events and other texts, I have borrowed the term *approximation* as the basis for my thinking about how certain texts construct a changeable, fluid ‘truth’ resulting from collisions, exchange and dialectical argument. The larger project from which this paper emanates,^1^ focuses on texts that are layered, and whose formal layerings mirror or augment a layered, multifaceted argument. Texts such as *Mad Men* are not bound by notions of closure and are suspicious of the idea of completeness—that an event or action can be finished, fixed and *not* open to reassessment. Films, programmes and media texts become *approximations* when they enact this suspicion in their structures; so frequently the dialectical arguments they pursue find echoes in the texts’ structures, layers of archive and other materials. At the heart of this symbiosis is a view of history, of the past as accessible and fluid—inevitably changing in relation to what follows. Like a series of tectonic plates, the elements that comprise approximations shift, overlap, collide and form different relationships with each other. What the notion approximation offers as a grounding concept for my thinking here, is the *mise*-*en*-*scene* or staging of fact and history: a place where what is known about a historical event, a factual occurrence, a real person is inserted into a film or narrative, not in order to be collapsed into fiction, but to co-exist in collision with it. *Approximations* are fact-based fantasies, stagings of evidence and fact, the re-enactments of the pooled resources of filmmakers, spectators, historians and other collators of evidence.

The specific example I will focus on in this paper is *Mad Men,* the brilliant, multiple Emmy award-winning US television series created by Matthew Weiner and transmitted by AMC, now in its seventh season. *Mad Men* is set in an advertising agency on New York’s Madison Avenue. The first season (2007) began right at the end of the 1950s and Season 7 has moved into 1969. Between each season roughly a year elapses, whilst each set of episodes travels through a relatively concentrated time period time. Time is important to *Mad Men* because, though fictional, the series remains, from the start, acutely conscious of the historical context it inhabits. The fictional characters live their lives against and respond to the real events that shaped American political history.

What interests me specifically in this discussion is the casual interjection of historical events into narratives that are otherwise entirely fictitious, a juxtaposition that *Mad Men* has always relished as it maps itself against not just the fashions, styles and social politics of the time but also momentous contemporary events, from the Nixon vs. Kennedy election of 1960 through the Cuban missile crisis, the murder of Medger Evers, the assassination of President John Fitzgerald Kennedy in Dallas, 22 November 1963 and later the assassinations of Martin Luther King and Robert Kennedy in 1968 (Season 6).

The consideration of *Mad Men* in the terms outlined above is a response to our current preoccupation with the diversification of the ways in which the media and related cultural forms represent, use and manipulate real events, especially against the backdrop of recent important technological advances. In this, the second decade of the 21st century, we are witnessing a particularly significant convergence of momentous historical events (of which the terrorist attacks of 11 September 2001 are the most notable) and equally momentous advances to our audiovisual media, an inevitable and welcome consequence of which is a global reassessment of how images are compiled, constructed, valued and received. The impact of ‘9/11’ (although it is also possible to argue that these events have become over-determined, their global significance over-stated) has been felt in a variety of ways; one way, it seems to me, has been to adopt a more relaxed attitude to elasticity and slipperiness when it comes to delineating the differences between ‘fact’ and ‘fiction’. For some critics, Oliver Stone’s editing together in *JFK* (1991), his film about the Kennedy assassination, of genuine archive and his own specially shot footage made to look like archive was a breach of trust; now, there is a greater acceptance of the frailties of such distinctions. As Baudrillard argued in the immediate aftermath of ‘9/11’, those attacks were ‘the “mother” of all events’ (2002: 3–4). Nearly everyone now knows to what the phrase ‘9/11’ refers, although there have been other 9/11s, such as the Chilean coup of 1973, led by General Augusto Pinochet. ‘9/11’ haunts what comes after it; as Susan Sontag famously commanded: ‘Let the atrocious images haunt us’. B. Ruby Rich, in the immediate aftermath of the attacks, considers the haunting slightly differently. Rich articulates the need to perceive the fruitfulness of distance and perspective slightly differently in an essay about teaching film after ‘9/11’. Rich writes how her ‘first impulse, faced with writing and teaching the events of 9/11 in a cinematic context’ was to go in search of Theodor Adorno’s infamous quotation ‘To write poetry after Auschwitz is barbaric’ (see Adorno 1967: 34; Rich 2004: 109). Her essay about 9/11 becomes, indirectly, an eloquent articulation of the problems of representing ‘the unrepresentable’.^2^ Having concluded that ‘in a very real sense, the events of 9/11 … have rendered inadequate the theoretical approaches and analytic habits on which film studies as a discipline has relied for the past several decades’ (109), Rich then identifies some of the texts she chose to teach, from the ‘totemic film’ *The Manchurian Candidate* (John Frankenheimer 1962) with its ‘eerie’ premonitions of the Kennedy assassination, to *The Battle of Algiers* (Gillo Pontecorvo 1966)—interesting because ‘in today’s context [it] has begun to look like a recruiting film for Al-Qaeda’ (111)—to Lizzie Borden’s *Born in Flames* (1983) in which ‘a tiny revolutionary cell of lesbian feminists blows up the top of the World Trade Center to destroy its lie-disseminating television transmitter after the government has assassinated the group’s leader’ (112). Links can be uncanny, fortuitous or contrived, but as Rich suggests, even random and tangential commentaries on subsequent historical events can prompt insights, can open up as opposed to narrow them down.

Within this overarching framework, my attention remains on factual and historical representation and, more specifically, what happens to the integrity of the original facts, documents and documentary at a time when the use of these fact-related forms by other media is altering our understanding of them completely. With the proliferation of DVD and the arrival of new, primarily internet-based viewing and distributing platforms, recognised, discrete categories such as ‘documentary’, ‘dramatisation’ and ‘fiction’ are being quite fundamentally and radically reassessed. In 1999, James M. Moran pondered the problem documentary theory faced from ‘the digital code’s circumvention of analogue recording’ (*Collecting Visible Evidence*, eds. Gaines and Renov: 267). Belief in the indexical properties of the factual analogue image has since been questioned (for example by myself in my book *New Documentary*), but the impact of the digital on how we interpret the authenticity of the factual image is only now being fully realised. In the digital age documents are available to be reworked, not just by filmmakers but also by viewers. A vital component of this engagement is the Internet. British filmmaker Adam Curtis, for example, has not only largely eschewed broadcast television in favour of putting his films out as stage performances or on the web (as he has done with *It Felt Like a Kiss* (2009) and *Bitter Lake* (2015), he has taken to composing short video essays or partaking in dialectical discussions with material garnered from the BBC archives on his blog (http://www.bbc.co.uk/blogs/adamcurtis). An alternative version of documentary’s loss of its indexical link to the real in the digital age has been the blurring of the boundaries between fact and fiction: UK newspaper *The Guardian*, for instance running, as part of its 2010 UK election coverage, the ‘Election briefings’ of the fictional character Malcolm Tucker, Peter Capaldi’s hysterical fictionalised caricature of Alastair Campbell from *The Thick of It* (BBC, 2005) and the film *In the Loop* (2009).

What is occurring, it seems to me, is an excitable flirtation with how to show and perform facts and evidence, with mixing genres and switching cultural arenas, the collective effect of which I want to explore through the concept of ‘approximation’, a term used in this context to signal works whose aim is to approximate reality rather than more straightforwardly represent it. Although the documents and facts on which ‘approximate’ texts are based remain pre-eminent, it is the detachment between the two that will remain my focus, as the various examples I want to examine in the larger project come from film, television and media-based art and are often not readily labelled either ‘fact’ and ‘fiction’.

This research is driven by the resulting dynamic relationship between raw documentary data (documents, archive, news and the like) and their re-use and repackaging in other, frequently fictional contexts. What ‘approximation’ offers is the *mise*-*en*-*scene* or staging of fact and history: a place where what is known about a historical event, a factual occurrence, a real person is inserted into a narrative, not in order to be collapsed into fiction, but to co-exist in collision with it. A point of departure for this conceptualisation of ‘approximation’ as a space of fantasy and desire is Elizabeth Cowie’s essay ‘Fantasia’ (*m/f,* 9, 1984) in which she argues (in relation to psychoanalysis and Hollywood film) that ‘the opposition real/not real is wholly inappropriate to a consideration of fantasy’ and that fantasy, usually ‘characterised as a series of wishes presented through imaginary happenings’ is also ‘a structure: fantasy as the *mise*-*en*-*scène* of desire, the putting into a scene, a staging, of desire’. In a comparable way, ‘approximations’ are stagings of evidence and fact, re-enactments of the pooled resources of filmmakers, spectators, historians and other collators of evidence. In *New Documentary* (2000, 2006) I argued that all documentary should be considered performative, a process of negotiation with the social world after Judith Butler in her book *Gender Trouble* (1990). I suggested that documentary film ‘is predicated upon a dialectical relationship between aspiration and potential, that the text itself reveals the tensions between the documentary pursuit of the most authentic mode of factual representation and the impossibility of that aim’ (Bruzzi 2006: 6–7). The crucial instability of documentary, I proposed, was the realisation that no documentary film could ever capture the performance of reality in front of the camera (80) and that all documentaries are ‘performative’ because they only come into being as they are performed. Although any film’s factual basis (or document) ‘can predate any recording or representation of it, the film itself is necessarily performative because it is given meaning by the interaction between performance and reality’ (186). ‘Approximations’ are propelled by the *frisson* of recognition: of knowing a film or drama’s point of reference, but also being able to recognise that the reconstruction and the point of reference are not equivalents. It is into this gap that we insert our desires, convictions and opinions.

I characterise approximation as a contemporary phenomenon. Although my intellectual starting point is the impact on representation as well as global politics of the terrorist attacks on the World Trade Centre in New York on 11 September 2001, ‘approximation’ is not simply ‘about’ 9/11, although these attacks offer a compelling example of how technology and events have conspired to make us reassess quite simply our ways of looking and seeing. Any such iconic event—the liberation of Bergen-Belsen, for instance, or the assassination of President Kennedy—has, in part come to be understood via the images that exist of it and the many far more elliptical allusions to it. In terms of 9/11: James Marsh’s documentary feature *Man on Wire* (2008), for instance can only now fully be understood in relation to and in recognition of these attacks; or the title sequence of *Mad Men* in which the monochrome man falling in front of a New York skyscraper can function semiotically as a visual metaphor for Don Draper, the series’ enigmatic and opaque central character, but also inevitably recalls the falling man who propelled himself from one of the burning towers on 9/11. As Janet Walker argues, ‘real catastrophes can disturb memory processing’ (*Trauma Cinema*, 2005: 4). Each iconic event throws into turmoil previous critical definitions of reality and representational forms, inviting us to reassess both the events’ importance and the images of it**—**its status as ‘icon’. In the context of ‘approximation’, event and mediated images of it cannot be ontologically separated, as the event is already, and perpetually iconic.^3^

There are not many historical events more iconic than the assassination of President Kennedy and we have just witnessed this during the spate of exhibitions, documentaries and feature films that commemorated the 50th anniversary of his death. I will here examine the insertion of this event into the penultimate episode of Series Three of *Mad Men*. As its audience realised that *Mad Men* would not only chart the lives of its fictional Madison Avenue characters but also map these on to American historical events, how the series would approach this traumatic, pivotal historical event became a topic for discussion. Season 2 had culminated with the Cuban Missile Crisis; Season 3 would inevitably reach November 1963.

I discussed the assassination of JFK in *New Documentary* as one of several iconic historical moments. By ‘iconic’ I meant traumatic events that had entered the collective memory and that possessed a specific amalgam of qualities: that they had been**—** *accidentally* **—**captured on camera (and so preserved in the image as well as in the memory) and they remained unexplained. Although we watch Abraham Zapruder’s 8 mm home movie footage of the assassination repeatedly to try and compel it to yield an explanation of what happened on 22 November 1963, as Bill Nichols comments, ‘To re-present the event is clearly *not* to explain it’ (Nichols 1994: 121). It is this central inadequacy that has led to a peculiar canonisation of certain emotionally charged pieces of film and video, such as the grainy Zapruder film, which yields, I would suggest, *the* iconic images of the Kennedy assassination. The images, fixed in time although perpetually open to reinterpretation, have become overly familiar fetish objects, even beautiful in their Kodak brightness, as the pink of Jackie’s suit clashes with the vivid green behind the presidential car. What is ironically crucial, however, to the impact of *Mad Men*’s rendition of 22 November 1963 is that, whilst *we* have the Zapruder images in our minds when watching it, this is precisely the footage that was certainly *not* available to view at the time of the assassination and wasn’t broadcast in its entirety for another decade. The Zapruder film has become the dominant assassination text, onto which is poured all the subsidiary grief, anger, belief in conspiracy and corruption surrounding the unresolved events it depicts. The text (an unedited, crude and ostensibly straightforward capturing of the assassination of Kennedy) appears simple enough, its meaning, however, is not, as it rapidly became the key source material for a multitude of conspiracy theories. Despite having the Zapruder film, we still do not know conclusively who killed JFK. As Roland Barthes observes, ‘Myth is not defined by the object of its message, but by the way in which it utters this message: there are formal limits to myth, there are no “substantial” ones’ (Barthes 1973: 117). Relating this to the Zapruder film: with each repeated viewing of it, do we get any closer to the myth it embodies? Do we simply see it for what it is**—**a piece of accidental amateur home movie footage? Or do we always see beyond the text to the wider ramifications of the death of Kennedy? The implications of Zapruder’s film are limitless, as the killing of President Kennedy is perpetually reworked.

*Mad Men* as a series is praised for its authentic though arch recreation—via costume, décor or office accessories, for example**—**of its early 1960s milieu. One thing it had to be was just as ‘authentic’ in its handling of the death of Kennedy, so the characters reconstruct the day’s television viewing as the news breaks: they happen upon the CBS Bulletins that interrupted the soap opera ‘As the World Turns’ and Walter Cronkite’s shocked announcement that the president has died. Although the characters do not follow the news on one channel but on several, the drama doesn’t deviate from a certain rule of authentication: the characters only watch what they could have watched on November 22 1963 and the days immediately after. I stress that for us, the later audience, what is emotionally important, however, is that we bring to our viewing of this sequence what has come out since: the capturing on 8 mm of Kennedy’s violent death, the conspiracy theories and that day’s effect on subsequent American history.

All these factors converge onto the re-watching of the ‘authentic’ television broadcasts—and necessarily alter our evaluations, perceptions of it. Like Americans at the time, the *Mad Men* characters are then glued to their television sets watching events unfold up to Kennedy’s funeral. The incidental nature of the treatment of Kennedy’s death is hugely significant: we have been waiting for the assassination to happen (we’ve known for a while, for instance, that Roger’s daughter is due to get married on 23 November 1963, the day after) but the characters have not. We know they are living through the most momentous event of 1960s American history, but the characters cannot be weighted down by this significance.

The television set plays the scene from ‘As the World Turns’ that many viewers, especially in the US, will recognise and which can now be re-viewed endlessly on *YouTube* being interrupted by the first CBS Bulletin, but only in the background—of a scene in which Peter tells a colleague how he didn’t get the promotion he’d hoped for. The extreme bathos of this moment is tonally complex: we are gearing up for a tragedy, but not one dependent upon the usual identification with on-screen characters. Earlier, I discussed ‘approximation’ as an excitable flirtation with how to show and perform facts and evidence, a desire to make us look differently at images and events that are often familiar by de-contextualising the factual source material. The frisson created by the approximate moments in *Mad Men* (and they share this with other such approximations)—what makes them especially moving—seems to me to be generated by these being moments when the audience knows more than the characters do and when the characters are trying to catch up. Steve Neale (after Franco Moretti 1986) determined that the most affecting moments in Hollywood melodramas—the scenes that finally brought one to tears—were those when a character who had been ignorant of something finally reaches the same point of knowledge and awareness as another character. Comparably, the affect, the ability to move of the approximate moment comes when fictional characters go someway towards reaching our point of knowledge and awareness about the factual events being re-stated. A hugely significant and moving factor in *Mad Men* is that we, the viewers, have been anticipating the death of JFK; this gearing up to what we know is an inevitable part of the fantasy, the emotive re-staging of what we already know and have assimilated. There is both comfort and shock in having the authentic original moment relived as it is in *Mad Men*; it does not necessarily add to our understanding of the events of 22 November 1963; its power resides in its ability to emotively invoke events that have become ingrained in our shared consciousness. Built into this is the melodramatic imperative of repetition, of revisiting an event that has already been deeply affecting—so the effect is multiplied.

The JFK episode raises key issues of approximation and shared consciousness by drawing on not only the familiar news footage from November 1963 but also our shared consciousness of what was not instantly available, notably the Zapruder film and the conspiracy theories. Coupled with the emotional intensity generated by the characters acquiring the same knowledge we bring to the approximate event, there is also the emotional intensity generated by knowing what, in terms of factual evidence, is being omitted. Observing the characters in *Mad Men* responding to the CBS Bulletins is even more affecting because in our imaginations their reactions trigger an imaginative recollection of all the information and images that, over time, we have acquired but they are still ignorant of.

The characters passing through history is a valued feature of *Mad Men* and in this instance time is both frozen (petrified in that one iconic moment) but also still marching on, as the characters continue into the future past the moment the world stopped or the dream died. In this, *Mad Men* is both a plausibly sincere rendition of what the day was like for ordinary Americans and a post-modern site on which all the accumulated knowledge about that day is brought together. The characters necessarily display a certain ‘innocence’ in their responses compared to our more cynical ‘experience’ of all the knowledge acquired since 22 November 1963, but just as it’s impossible to return to William Blake’s *Songs of Innocence* and read them as innocent (as opposed to maybe fraudulently naïve) after having read the twin volume *Songs of Experience,* so our cynicism stains the authentic spontaneity of this incidental approximate moment. *Mad Men* is rooted in the collision between memory and authenticity, so we evaluate the series’ dramatised mimesis of the events of that day via our own memories. The series is also a *re*-mediation of an already mediated memory: ‘mediated’ because the events of Dealey Plaza Dallas, November 1963, are for the vast majority of us ingrained as images. I recently went to Dealey Plaza and was struck by how being there at the site of this iconic unsolved crime was an example of what can most usefully be termed the *‘reverse* uncanny’: visiting a location that is familiar or iconic through *images*, images that are subsequently repeated as sources for narratives, histories and memories and come to substitute for the events themselves. Visiting an iconic location compels us to re-live in our minds and with our gestures not the events themselves but their perpetual re-mediation.

*Conclusion* ‘Approximation’ is characterised by layering, by texts which tacitly assume strata of awareness that eventually lead us back to core iconic antecedents that perhaps no longer need to be named directly in order to be evoked. I started by suggesting that our attraction to these approximations is more prevalent now and linked to the multiplication of the ways in which images can be generated and consumed. The ways we look at reality are changing as rapidly as the world we live in; we are thus, in an example such as *Mad Men,* being offered new ways of looking at and conceptualising the document, the factual image, which in turn is helping to shape the cultural landscape of the 21st century.

## Footnotes

1
*Approximation: Documentary, History and the Staging of Reality* is a 24-month Major Research Fellowship, which received generous funding from the Leverhulme Trust, UK. More details can be found on the project’s website: http://www.approximation.org.uk/.

2 See for example Thomas Elsaesser (2001) and many previous discussions of the Holocaust and representation.

3 I would like to thank Frederik Le Roy for crucially pointing out that the iconic event in this context is always already mediated.
